# Foot Biomechanics, Body Composition, and Sex‐Related Factors Associated With Work‐Related Musculoskeletal Discomfort in Standing Assembly‐Line Workers: A Cross‐Sectional Study

**DOI:** 10.1002/jfa2.70233

**Published:** 2026-10-27

**Authors:** Alejandro‐Jesús Almenar‐Arasanz, José‐Antonio Villalba‐Ruete, Elisa Bravo‐Viñuales, Ana Vanessa Bataller‐Cervero, Juan Rabal‐Pelay, Jacobo de‐Rentería‐de‐la‐Peña, Luis‐Enrique Roche‐Seruendo, Cristina Cimarras‐Otal

**Affiliations:** ^1^ Universidad San Jorge Zaragoza Spain; ^2^ BSH‐EE Zaragoza Spain; ^3^ Hospital MAZ Zaragoza Spain

**Keywords:** 3D foot scanning, assembly‐line workers, baropodometry, foot posture, plantar pressure, prolonged standing, work‐related musculoskeletal disorders

## Abstract

**Background:**

Prolonged standing is common in manufacturing and assembly‐line work and is associated with musculoskeletal discomfort, especially in the lower limbs and spine. The influence of foot posture, morphology, and plantar loading patterns on this discomfort remains unclear.

**Objective:**

To analyze the associations between foot posture, plantar pressure, and 3D foot morphology with work‐related musculoskeletal discomfort in standing workers and to examine the role of BMI and the potential modifying role of sex.

**Methods:**

A cross‐sectional study was conducted among 240 assembly‐line workers (129 women, 53.8%). Musculoskeletal discomfort was assessed using the Cornell Musculoskeletal Discomfort Questionnaire (CMDQ). Foot posture (FPI‐6), baropodometry (FreeMed), and 3D foot morphology (DOMESCAN) were evaluated under standardized conditions. Participants were classified as having low (CMDQ ≤ 10) or moderate–high (≥ 11) discomfort. Firth‐penalized logistic regression analyzed associations between overall CMDQ discomfort, foot posture, sex, and BMI, including an FPI × sex interaction term.

**Results:**

All workers reported at least one musculoskeletal discomfort response, with the feet, neck, and lumbar region accounting for the largest proportions of reported discomfort. Among women, pronated foot posture was associated with higher odds of moderate–high overall CMDQ discomfort (OR = 2.91, 95% CI 1.10–7.28). Overweight/obesity status was associated with higher odds of foot and hip/gluteal discomfort, whereas, among women, pronated foot posture was associated with greater odds of lumbar discomfort. The pronation–discomfort association was stronger in women and attenuated in men.

**Conclusions:**

Among women, pronated foot posture was associated with greater overall work‐related musculoskeletal discomfort during prolonged standing and with greater odds of lumbar discomfort, whereas overweight/obesity status was associated with greater odds of foot and hip/gluteal discomfort. The pronation–discomfort association was attenuated in men. From an occupational‐health perspective, incorporating foot‐posture and BMI assessment into ergonomic evaluations may help identify workers who could benefit from more individualized preventive strategies, particularly women with pronated feet.

## 1 Introduction

Work‐related musculoskeletal disorders (WMSDs) are the most prevalent occupational diseases worldwide and are responsible for a substantial proportion of long‐term sick leave and work‐related disability across sectors [1, 2]. In Spain, WMSDs remain a significant public health issue within the occupational setting. According to the most recent data from the 2021 European Working Conditions Survey, 87.2% of Spanish workers reported at least one health problem in the past 12 months, with musculoskeletal disorders being the most frequent. Specifically, 63.9% reported back pain, 65.4% reported muscular pain in the shoulders, neck, and upper limbs, and 40.5% reported discomfort in the lower limbs, including hips, legs, knees, and feet [3]. These patterns are particularly concerning in assembly line workers, who are exposed to static postures, limited gait variability, and sustained biomechanical loading [4, 5].

Assembly line workers typically perform their tasks standing for prolonged periods, often with limited mobility and repetitive upper limb activity [3, 6]. Prolonged standing is a recognized risk factor for discomfort and pain in the feet, knees, and lumbar region, with studies reporting up to 48% of workers experiencing foot and ankle discomfort [7]. The Canadian Center for Occupational Health and Safety similarly highlights back pain, sore feet, varicose veins, and general fatigue as the primary complaints associated with extended periods of standing [8].

Given the biomechanical demands of prolonged standing, increasing attention has been directed toward the role of foot structure and function in the development of musculoskeletal disorders, particularly due to their influence on load distribution throughout the lower limbs and spine [4, 5]. Both excessively pronated (planus) and supinated (cavus) foot types have been linked to discomfort and injury in the ankle, knee, and lumbar spine, as altered alignment can affect joint mechanics and muscle activation patterns [9, 10].

One of the most reliable tools for classifying static foot alignment is the Foot Posture Index (FPI‐6) [11]. However, recent studies suggest that the FPI alone may not fully capture dynamic load behavior during gait, supporting the value of combining it with baropodometric and morphological analyses [10, 12]. Baropodometric assessment enables the quantification of plantar pressure patterns under static and dynamic conditions [12]. For instance, patients with metatarsalgia exhibit reduced forefoot loading and increased rearfoot pressure, often with cavus posture, while those with plantar fasciitis display elevated forefoot pressures and a pronated profile [13]. In occupational contexts, prolonged static weight‐bearing has also been associated with increased plantar contact surface and discomfort, even when total pressure remains stable [7].

Foot morphology, including arch height, width, and plantar surface area, also influences biomechanical efficiency and tissue loading [14]. Flexible flatfoot has been associated with altered strain patterns in the plantar fascia, increasing the risk of chronic heel or arch pain in tasks requiring repetitive movement and static posture [14]. Recent advances in technology have introduced three‐dimensional (3D) scanners as precise, non‐invasive tools for quantifying foot morphology, including arch structure, instep volume, and plantar surface geometry under weight‐bearing conditions [15]. Their implementation in occupational health research represents a novel and objective approach that enhances the reliability of morphological assessment beyond traditional visual or palpatory methods [15].

Individual characteristics such as BMI and sex may also be relevant to musculoskeletal discomfort in workers exposed to prolonged standing. Higher BMI increases mechanical loading on the lower limbs, and prospective evidence in working populations has associated elevated BMI with severer lower‐limb musculoskeletal symptoms [16]. Sex‐related differences may also be relevant, as previous population‐based research has reported an association between pronated foot function and lumbar pain in women [17]. These findings support considering BMI and sex alongside foot biomechanics when examining musculoskeletal discomfort.

Identifying musculoskeletal discomfort in occupational environments requires validated tools that evaluate multiple anatomical regions. The Cornell Musculoskeletal Discomfort Questionnaire (CMDQ) is widely used for this purpose, assessing the frequency, intensity, and work interference of discomfort in areas such as the neck, back, and upper and lower limbs [18]. The CMDQ has demonstrated high internal consistency and reliability in diverse industrial contexts, including Spanish‐speaking populations [7, 18, 19]. By linking subjective symptom reports with objective exposure factors, its integration with biomechanical variables such as foot posture, plantar pressure, and morphology provides a multidimensional approach to identifying workers at risk of WMSDs [7, 18, 19].

Although prolonged standing is recognized as a risk factor for musculoskeletal discomfort, the underlying biomechanical mechanisms remain insufficiently understood. Most studies have examined isolated factors such as posture or workstation design, without integrating the mechanical role of the foot, which acts as a key structure for postural control and load transfer during static conditions [4, 7]. Only a few investigations have explored the relationship between foot posture, plantar pressure distribution, and morphology with validated symptom‐reporting instruments such as the CMDQ, particularly in real industrial populations [7, 18, 19]. To the best of the authors' knowledge, no previous studies have examined the relationship between foot type as assessed by the FPI‐6, 3D foot morphology, plantar pressure distribution and the musculoskeletal discomfort experienced by workers during their workday.

Therefore, this study aimed to (i) describe the anatomical distribution of discomfort, (ii) analyze biomechanical differences in foot posture, baropodometry and 3D foot morphology according to discomfort level, and (iii) identify the biomechanical and morphological foot factors associated with overall and region‐specific musculoskeletal discomfort, examine the role of BMI, and assess the potential modifying role of sex in standing assembly‐line workers. We hypothesized that workers exposed to prolonged standing would present measurable associations between foot posture, plantar pressure distribution, and self‐reported musculoskeletal discomfort, and that BMI and sex would be associated with discomfort, with sex potentially modifying the association between foot posture and discomfort.

## 2 Material and Methods

### 2.1 Study Design and Setting

This study was designed as a cross‐sectional descriptive investigation. Data collection was carried out at the BSH Montañana industrial plant (Zaragoza, Spain), a manufacturing facility dedicated to the production of cooking appliances, between May and November 2024, in a designated space near the assembly line to minimize disruption to workers while ensuring privacy and standardized measurement conditions. This study was conducted following the recommendations of the STROBE (Strengthening the Reporting of Observational Studies in Epidemiology) statement for cross‐sectional studies.

### 2.2 Participants

Participant recruitment was coordinated through the company's medical service. Eligible workers were identified from internal records and contacted via corporate email. Each potential participant received an information sheet outlining the study objectives and procedures, along with a secure link to an online informed consent form and the Cornell Musculoskeletal Discomfort Questionnaire (CMDQ). Participation was voluntary, and individuals not meeting inclusion criteria or declining to participate were excluded from data collection.

Inclusion criteria were: age between 18 and 65 years, current employment in a standing assembly line position (8 hours work‐day), absence of medical restrictions that could affect task performance, and provision of informed consent [4, 6, 7].

Exclusion criteria included: prior surgery of the feet or significant lower‐limb or spinal injuries or disorders limiting mobility; neurological or rheumatologic conditions affecting gait or posture; pregnancy; or inability to complete the CMDQ or biomechanical assessments [4, 7, 10, 16].

Workers followed a fixed morning shift from 06:00 to 14:00 h, which included a 15‐minute break for breakfast and any additional rest periods established by company policy.

### 2.3 Sample Size Calculation

The required sample size for a finite population (*N* = 988) was computed assuming a 95% confidence level (*z* = 1.96), a 5% margin of error, and an expected prevalence of musculoskeletal discomfort of 30% based on prior reports in manufacturing workers exposed to prolonged standing [20, 21]. Applying the finite population correction yielded a target sample size of approximately 240 participants.

### 2.4 Ethical Considerations

This study adhered to the Declaration of Helsinki and was approved by the Research Ethics Committee of Aragón (CEICA) under protocol PI24/176. All participants were informed verbally and in writing about the study aims, procedures, and voluntary nature of participation. Electronic informed consent was obtained prior to data collection. Confidentiality and anonymity were ensured; all data were pseudonymized before analysis.

### 2.5 Use of Artificial Intelligence

Parts of this manuscript benefited from language editing and text refinement using ChatGPT (OpenAI, San Francisco, USA) under full human oversight. No artificial intelligence tools were used to generate, analyze, or interpret data, nor to develop the study design, methods, results, or conclusions.

### 2.6 Instruments and Assessment Protocol

All measurements were performed on‐site at the BSH Montañana industrial plant (Zaragoza, Spain) in a designated area adjacent to the production line to ensure privacy, minimize interference with work activity, and maintain standardized testing conditions. Assessments were conducted between 9:00 and 14:00 h during the workers' regular shift hours. Each evaluation lasted approximately 15–20 minutes and was carried out by an experienced physiotherapist (> 5 years of clinical and research experience in biomechanics), assisted by a second researcher responsible for data management and quality control. All data were pseudonymized prior to analysis.

- Anthropometry: Body weight and height were measured with participants barefoot using a Tanita MC‐780MA analyzer (Tanita Corporation, Japan) and a SECA stadiometer (model 206, Seca Corp., Hanover, MD, USA; precision 1 mm), respectively. Participants stood barefoot in a relaxed bipedal stance and their heads aligned in the Frankfort horizontal plane, following ISAK guidelines [22].
- Musculoskeletal discomfort: Musculoskeletal discomfort was evaluated using the Cornell Musculoskeletal Discomfort Questionnaire (CMDQ), completed online before the on‐site assessment. This validated instrument assesses 20 anatomical regions, recording the frequency, intensity, and interference of discomfort experienced during the previous workweek. A composite score for each region was obtained as the product of frequency (0–10), discomfort [1, 2, 3], and interference [1, 2, 3] ratings, yielding regional scores from 0 to 90, following the Cornell Human Factors and Ergonomics Research Group guidelines [23]. Total CMDQ scores were calculated by summing regional values. The CMDQ has demonstrated high internal consistency and validity in industrial and Spanish‐speaking populations [7, 18, 19].
- Foot posture: Foot alignment was assessed using the Foot Posture Index (FPI‐6), a validated clinical instrument based on visual and palpatory examination [11]. The FPI‐6 comprises six items (talar head palpation, malleolar curvature, calcaneal frontal position, talonavicular prominence, medial arch height/congruence, and forefoot abduction/adduction), each scored from −2 to +2, with total values ranging from −12 (maximum supination) to +12 (maximum pronation). The index was applied only to the right foot, based on evidence of minimal bilateral differences in asymptomatic adults [24]. Evaluations were performed with the participant standing barefoot and executed by a single trained physiotherapist to ensure intra‐evaluator consistency. The assessment was performed visually and by palpation, following standard procedures.
- Plantar pressure analysis: Static and dynamic plantar pressure measurements were obtained using the FreeMed capacitive platform (Sensor Medica, Italy), which records high‐resolution baropodometric data.
  ◦ Static test: Participants stood barefoot in a relaxed bipedal stance for 20 seconds, with feet shoulder‐width apart, toes forward, arms relaxed at the sides, and head facing forward, replicating typical workplace posture [7, 12].
  ◦ Dynamic test: Participants walked at a comfortable, self‐selected pace, following the validated three‐step protocol [7, 12, 21]. The third step, landing entirely on the sensing surface, was used for analysis to avoid acceleration/deceleration effects. A minimum of three consistent trials were recorded.

The platform generated multiple pressure‐related variables (total, regional, and temporal). Among them, hallux load (percentage of total plantar load borne by the hallux) and rocker timings, i.e., initial contact phase (ICP), forefoot contact phase (FFCP), and forefoot propulsion phase (FFP), were selected for reporting given their biomechanical relevance. The FreeMed system, based on capacitive sensor technology, has demonstrated excellent repeatability and reliability in both static and dynamic assessments [12]. Data were acquired and processed using the FreeStep v.1.0.3 software (Sensor Medica, Italy).

- 3D foot morphology: Foot geometry was captured using the DOMESCAN 3D foot scanner (Instituto de Biomecanica de Valencia, Spain), a non‐invasive, high‐precision optical system that records the three‐dimensional structure of the foot under static weight‐bearing conditions. Scanning was performed on the right foot only, justified by bilateral morphological symmetry and the operational need to streamline data collection without compromising accuracy [15, 24]. The scanner provided numerous morphological parameters; foot length, width, arch height and area, navicular height, plantar surface area, and podalic angle (foot progression angle) were retained for analysis. All variables were normalized to foot length unless otherwise stated [10, 25]. DOMESCAN has shown excellent accuracy and reproducibility in clinical and ergonomic research [14].

### 2.7 Statistical Analysis

Continuous variables were inspected with the Shapiro–Wilk test for normality and Levene's test for homoscedasticity. Normally distributed data are expressed as mean ± standard deviation (SD), and non‐normally distributed variables as median and interquartile range (IQR, 25th–75th percentile). Categorical variables are shown as *n* (%).

CMDQ regional scores were computed as the product of frequency × discomfort × interference and summed across all 20 regions to obtain total CMDQ values [23]. The original CMDQ scoring guidance and the Spanish‐language validation study do not establish validated clinical cut‐off values for the total CMDQ score [19, 23]. Therefore, study‐specific analytical thresholds were used to facilitate interpretation of the score distribution: Low (≤ 10) and Moderate–High (≥ 11) for the primary analyses, with an additional High threshold (≥ 31) used for sensitivity analyses. These categories were intended solely for analytical stratification and should not be interpreted as validated clinical severity cut‐offs. For anatomical distribution analyses, regional discomfort reports were summed within each CMDQ group and expressed as a percentage of all regional discomfort reports in that group, with each group normalized to 100%. Therefore, these percentages represent the relative anatomical distribution of reported discomfort and not region‐specific prevalence among workers.

Group comparisons were performed using *t*‐tests or Mann–Whitney *U* tests, as appropriate, with statistical significance set at *p* < 0.05 (two‐sided). Adjusted *p*‐values were derived from ordinary least‐squares (OLS) models with HC3 robust errors, controlling for sex and BMI category (normal weight 18.5–24.9 kg/m^2^; overweight/obese ≥ 25 kg/m^2^ per WHO criteria [26]). Effect sizes were reported as Cohen's *d* or rank‐biserial *r*, and interpreted as small (*d* = 0.2), medium (*d* = 0.5), and large (*d* = 0.8) [27].

A broad set of morphological and plantar pressure variables was examined; only significant or biomechanically relevant parameters (*p* < 0.10) are presented in the main tables, with the complete list in Table S1. Multivariable associations between discomfort (Moderate–High vs. Low) and FPI, sex and BMI, including an FPI × sex interaction term, were tested using Firth‐penalized logistic regression, reported as odds ratios (OR) with 95% confidence intervals (CI). Because some combinations of FPI category and sex contained relatively few observations, Firth‐penalized logistic regression was used to reduce small‐sample and separation‐related bias.

Among participants with complete CMDQ and biomechanical data, observations with an absolute *z*‐score > 3 for any continuous biomechanical variable were considered outliers and excluded from the primary analysis (*n* = 6). Sensitivity analyses including these observations did not materially alter the main findings. All analyses were performed using Python 3.11.8 (Python Software Foundation, Wilmington, DE, USA).

## 3 Results

A total of 283 direct labor workers were initially recruited for the study. Of these, 37 participants were excluded for not completing the CMDQ. Of the 246 participants with complete CMDQ and biomechanical data, six met the outlier criterion (> 3 SD for at least one continuous biomechanical variable) and were excluded, yielding a final analytical sample of 240 participants. The final sample consisted of 240 standing assembly line workers, all of whom met the inclusion criteria and provided complete and valid data for analysis (Figure 1).

**FIGURE 1:**
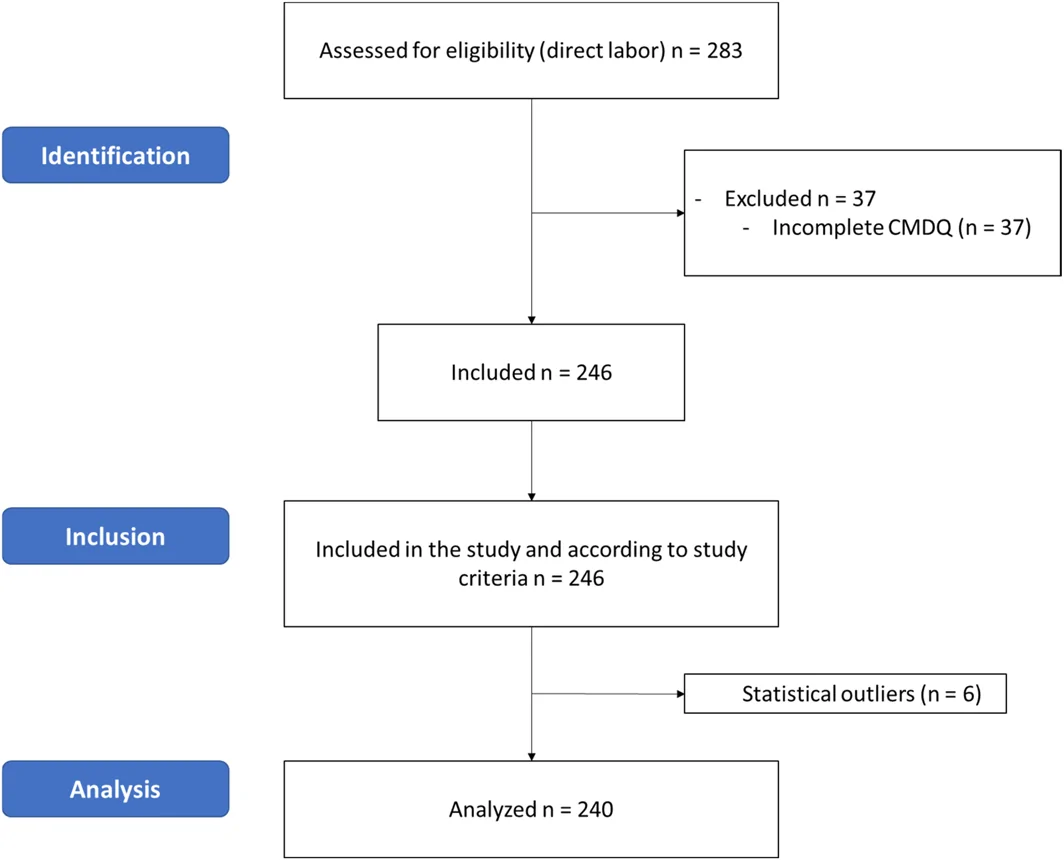
STROBE flow chart. STROBE, strengthening the reporting of observational studies in epidemiology.

All participants reported at least one musculoskeletal discomfort response in the CMDQ, although some participants had a total composite score of zero because of the multiplicative scoring procedure. Study‐specific analytical CMDQ thresholds based on the total discomfort score were applied to facilitate interpretation. The primary grouping contrasted Low (CMDQ ≤ 10) versus moderate–high (CMDQ ≥ 11) discomfort for descriptive summaries and multivariable models. Because CMDQ distributions are inherently right‐skewed and intermediate scores may dilute contrasts, an additional extreme‐group comparison (Low ≤ 10 vs. High ≥ 31) was performed as a sensitivity analysis to maximize biomechanical differentiation. This two‐tier analytical approach, based on the global CMDQ score, improves interpretability while accounting for the distributional characteristics of discomfort data.

### 3.1 Overall Characteristics and Primary Grouping

Based on the global CMDQ score, which reflects overall work‐related discomfort across all body regions, 76.7% of workers were classified as Low (CMDQ ≤ 10) and 23.3% as moderate–high (CMDQ ≥ 11). As shown in Table 1, both groups were comparable to age, sex, anthropometrics, and work experience. The Foot Posture Index (FPI‐6) showed a predominance of neutral posture (≈ 53%), with pronated (≈ 27%) and supinated (≈ 20%) feet distributed similarly between groups. These results indicate that lower CMDQ scores predominated, while about one quarter of workers were classified in the study‐specific moderate–high analytical category.

**TABLE 1:** Demographic and occupational characteristics of participants according to musculoskeletal discomfort level (*n* = 240).

Variable	Low discomfort (*n* = 184)	Moderate–High discomfort (*n* = 56)	Total (*n* = 240)
Age (years)	47.6 ± 6.2	49.0 ± 7.1	47.9 ± 6.4
Height (cm)	169.3 ± 8.7	169.3 ± 9.0	169.3 ± 8.7
Weight (kg)	73.6 ± 15.6	75.4 ± 13.7	74.0 ± 15.2
BMI (kg/m^2^)	25.6 ± 4.8	26.2 ± 3.7	25.7 ± 4.6
Normal weight (*n* (%))	101 (54.9%)	22 (39.3%)	123 (51.3%)
Overweight (*n* (%))	83 (45.1%)	34 (60.7%)	117 (48.8%)
Years of work experience	20.1 ± 7.9	22.6 ± 7.9	20.7 ± 7.9
Shoe size (EU)	40.6 ± 2.7	41.0 ± 2.7	40.7 ± 2.7
Sex (*n* (%))			
Female	99 (53.8%)	30 (53.6%)	129 (53.8%)
Male	85 (46.2%)	26 (46.4%)	111 (46.2%)
Foot posture index (FPI) (*n* (%))			
Neutral	100 (54.3%)	28 (50.0%)	128 (53.3%)
Supinated	36 (19.6%)	12 (21.4%)	48 (20.0%)
Pronated	48 (26.1%)	16 (28.6%)	64 (26.7%)
Total CMDQ score	0.6 ± 0.8 [0.6 (0–3.6)]	24.1 ± 13.6 [18.3 (12.9–31.3)]	7.5 ± 11.6 [2.8 (0–14.2)]

*Note:* Continuous variables are expressed as mean ± standard deviation (SD) [median (interquartile range, IQR)], and categorical variables as frequency (percentage). Discomfort classification based on study‐specific analytical thresholds of the CMDQ total score: Low (≤ 10) versus Moderate–High (≥ 11).

Abbreviations: BMI, body mass index; CMDQ, Cornell Musculoskeletal Discomfort Questionnaire; FPI, Foot Posture Index; IQR, interquartile range; SD, standard deviation.

### 3.2 Anatomical Distribution of Discomfort

Figure 2 shows the relative anatomical distribution of reported discomfort within each CMDQ group, rather than region‐specific prevalence. In the Low group, the largest proportions of reported discomfort corresponded to the feet (23.4%), neck (18.6%), and lumbar region (16.0%). In the moderate–high group, the same pattern was observed, with the feet (15.4%), neck (13.4%), and lumbar region (12.4%) accounting for the largest proportions.

**FIGURE 2:**
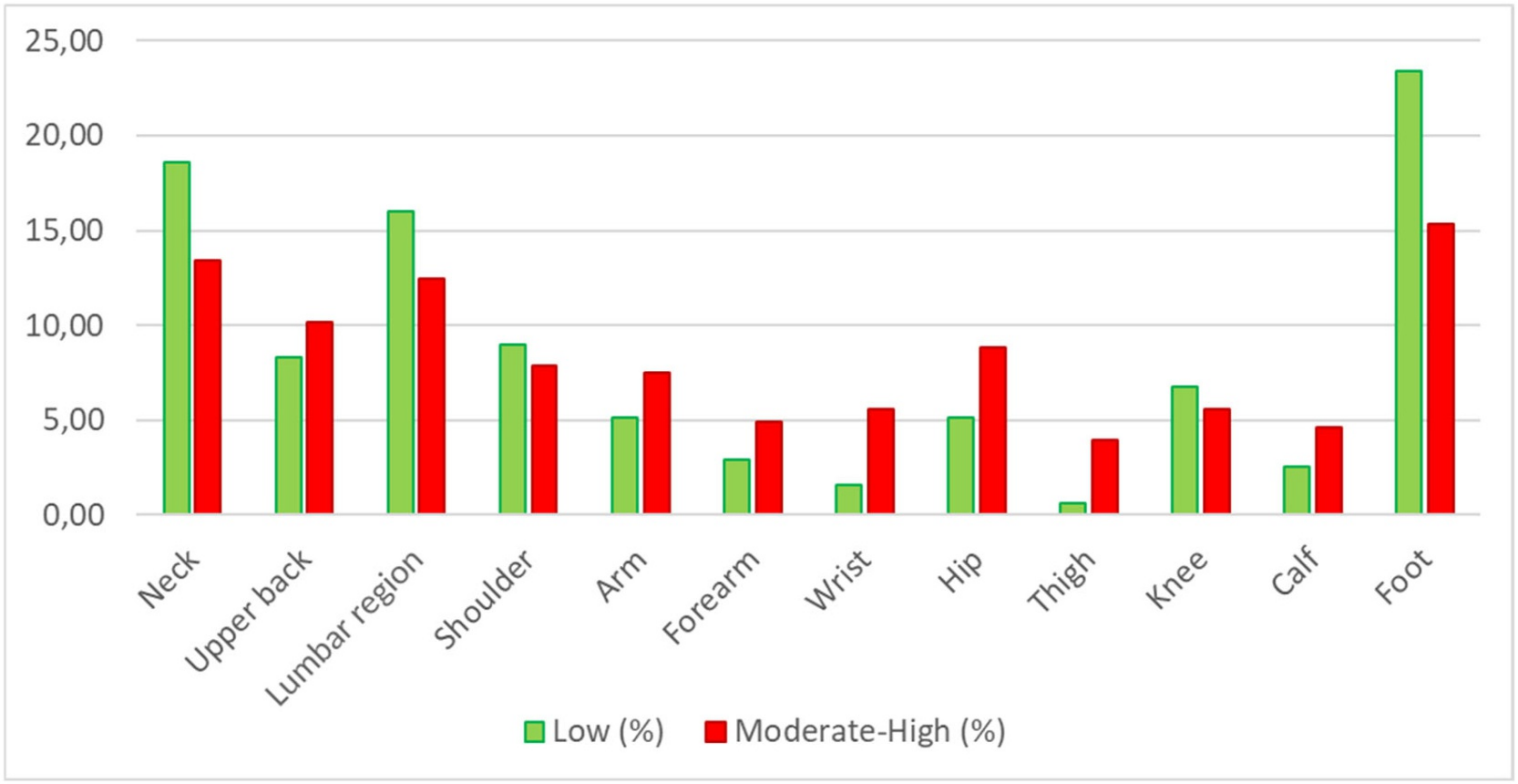
Anatomical distribution of discomfort across CMDQ groups. Relative frequency of discomfort by anatomical region among workers was classified into low and moderate–high discomfort groups according to the Cornell Musculoskeletal Discomfort Questionnaire (CMDQ). Each group was normalized to 100%. Percentages represent the relative distribution within each group and should not be interpreted as region‐specific prevalence.

### 3.3 Extreme‐Groups Descriptive Contrast (Sensitivity Analysis)

To enhance biomechanical contrast, a sensitivity analysis compared the Low (CMDQ ≤ 10) and High (CMDQ ≥ 31) discomfort groups in participants with complete biomechanical data (*n* = 198; Low = 184, High = 14). Intermediate cases were excluded to reduce variability from mid‐range scores, as outlined in the Methods. As shown in Table 2, the two groups showed similar demographic and anthropometric profiles, suggesting that potential biomechanical differences are unlikely to be attributable to these factors. This contrast aimed to enhance biomechanical differentiation while acknowledging the limited representativeness of extreme categories.

**TABLE 2:** Descriptive characteristics of participants according to extreme discomfort categories (*n* = 198).

Variable	Low discomfort (*n* = 184)	High discomfort (*n* = 14)	Total (*n* = 198)
Age (years)	47.6 ± 6.2	48.5 ± 8.4	47.7 ± 6.3
Height (cm)	169.3 ± 8.7	167.9 ± 8.6	169.2 ± 8.7
Weight (kg)	73.6 ± 15.6	72.2 ± 14.8	73.5 ± 15.6
BMI (kg/m^2^)	25.6 ± 4.8	25.4 ± 3.7	25.6 ± 4.7
Normal weight (*n* (%))	101 (54.9%)	8 (57.1%)	109 (55.1%)
Overweight (*n* (%))	83 (45.1%)	6 (42.9%)	89 (44.9%)
Years of work experience	20.1 ± 7.9	21.4 ± 7.9	20.2 ± 7.9
Shoe size (EU)	40.6 ± 2.7	40.8 ± 2.8	40.7 ± 2.7
Sex (*n* (%))			
Female	99 (53.8%)	7 (50.0%)	106 (53.5%)
Male	85 (46.2%)	7 (50.0%)	92 (46.5%)
Foot posture index (FPI) (*n* (%))			
Neutral	100 (54.3%)	9 (64.3%)	109 (55.1%)
Supinated	36 (19.6%)	4 (28.6%)	40 (20.2%)
Pronated	48 (26.1%)	1 (7.1%)	49 (24.7%)
Total CMDQ score	0.6 ± 0.8 [0.6 (0–3.6)]	43.3 ± 9.6 [41.9 (35.7–48.5)]	5.4 ± 11.5 [0.6 (0–4.7)]

*Note:* Continuous variables are expressed as mean ± standard deviation (SD) [median (interquartile range, IQR)], and categorical variables as frequency (percentage). Extreme‐group classification based on CMDQ total score: Low (≤ 10) versus High (≥ 31).

Abbreviations: BMI, body mass index; CMDQ, Cornell Musculoskeletal Discomfort Questionnaire; FPI, Foot Posture Index; IQR, interquartile range; SD, standard deviation.

### 3.4 Bivariate Associations in the “Extreme” Contrast (Low vs. High)

Among the full set of morphological and plantar pressure parameters, only those showing statistically or biomechanically relevant contrasts are reported in Table 3. The podalic angle was greater in the High discomfort group (12.93 ± 4.03° vs. 10.14 ± 4.48°; *p* = 0.003; Cohen's *d* = 0.63), indicating a moderate effect size. Normalized heel width differed modestly (0.26 ± 0.01 vs. 0.26 ± 0.01; *p* = 0.013; *d* = 0.39, small to moderate), and indices of arch area showed borderline differences (normalized arch area 0.05 ± 0.01 vs. 0.04 ± 0.01, *p* = 0.050; segmented arch area 0.14 ± 0.02 vs. 0.12 ± 0.03, *p* = 0.055). The initial contact phase (ICP) tended to be shorter in the High discomfort group (128.8 ± 60.0 ms vs. 163.6 ± 67.5 ms; *p* = 0.081), while hallux load was slightly lower, not higher, in that group (9.70 ± 4.48% vs. 11.90 ± 4.37%; *p* = 0.143; *d* = −0.50). Although not all differences reached statistical significance, the moderate effect sizes observed suggest potentially relevant biomechanical contrasts.

**TABLE 3:** Comparison of foot morphological and plantar pressure variables between workers with High (CMDQ ≥ 31) and Low (≤ 10) discomfort (extreme‐groups contrast; *n* = 198).

Variable	Low discomfort (mean ± SD)	High discomfort (mean ± SD)		Adjusted *p* (OLS HC3; sex, BMI, FPI)	Effect size
Weight (kg)	73.56 ± 15.64	72.25 ± 14.80		0.24^a^	0.005
Height (cm)	169.32 ± 8.67	167.86 ± 8.65		0.345^b^	−0.169
BMI (kg/m^2^)	25.60 ± 4.83	25.44 ± 3.66		0.355^a^	0.035
Age (years)	47.62 ± 6.19	48.46 ± 8.36		0.734^b^	0.131
Shoe size (EU)	40.65 ± 2.72	40.79 ± 2.77		0.989^a^	0.009
Podalic angle (°)	10.14 ± 4.48	12.93 ± 4.03		**0.003** ^b^	0.626
Normalized heel width	0.26 ± 0.01	0.26 ± 0.01		**0.013** ^b^	−0.388
Normalized arch area	0.04 ± 0.01	0.05 ± 0.01		**0.05** ^a^	0.129
Segmented arch area	0.12 ± 0.03	0.14 ± 0.02		0.055^a^	0.134
Rockers ICP (ms)	163.59 ± 67.52	128.79 ± 60.03		0.081^a^	0.131
Hallux load (%)	11.90 ± 4.37	9.70 ± 4.48		0.143^b^	−0.503

*Note:* Values are mean ± SD. Adjusted *p*‐values from OLS models with HC3 robust SE (controlled for sex, BMI, and FPI). Effect sizes are reported as Cohen's *d* for Student's *t*‐tests and rank‐biserial *r* for Mann–Whitney *U* tests (Cohen's d: small = 0.2, medium = 0.5, large = 0.8). Significant results in bold.

Abbreviations: BMI, body mass index; CMDQ, Cornell Musculoskeletal Discomfort Questionnaire; FPI, Foot Posture Index; HC3, heteroskedasticity‐consistent type 3 robust standard errors; ICP, initial contact phase; OLS, ordinary least squares; SD, standard deviation.

^a^ Mann–Whitney *U* test.

^b^ Student's *t*‐test.

### 3.5 Multivariable Model for Overall Discomfort (Primary Analysis)

Among women, the reference sex in the model, pronated foot posture was associated with higher odds of moderate–high overall discomfort compared with neutral foot posture (OR = 2.91, 95% CI: 1.10–7.28). Overweight showed a positive but non‐definitive association (OR = 1.78, 95% CI: 0.93–3.61). Among participants with neutral foot posture, the main effect for male sex was not statistically significant (OR = 1.52, 95% CI: 0.68–3.52). The pronated foot posture × male sex interaction was below 1 (OR = 0.19, 95% CI: 0.04–0.73), indicating that the association between pronation and discomfort was attenuated in men compared with women. Supinated foot posture and its interaction with sex were not statistically significant (Table 4).

**TABLE 4:** Firth‐penalized logistic regression model for overall musculoskeletal discomfort (CMDQ total).

Model term	OR	95% CI (lower)	95% CI (upper)
Intercept (reference: neutral foot posture, female, normal weight)	0.42	0.30	0.60
Pronated foot posture	**2.91**	1.10	7.28
Supinated foot posture	1.43	0.30	3.90
Male sex	1.52	0.68	3.52
Overweight	1.78	0.93	3.61
Pronated × male sex	**0.19**	0.04	0.73
Supinated × male sex	0.68	0.15	3.55

*Note:* Results are presented as odds ratios (OR) with 95% confidence intervals (CI). The dependent variable was moderate–high discomfort (CMDQ ≥ 11) versus Low discomfort (≤ 10) (*n* = 240). Independent variables included foot posture (FPI categories: pronated, supinated, neutral), sex, BMI classification (normal weight vs. overweight/obese), and the interaction term FPI × sex. Firth penalized likelihood was used to reduce small‐sample and separation‐related bias in sparse subgroup combinations. Because female sex was the reference category, the main‐effect ORs for FPI represent the association between each foot‐posture category and discomfort among women; the FPI × sex interaction terms indicate how these associations differ in men. Statistically significant model terms are highlighted in bold.

Abbreviations: BMI, body mass index; CI, confidence interval; CMDQ, Cornell Musculoskeletal Discomfort Questionnaire; FPI, Foot Posture Index; OR, odds ratio.

Firth‐penalized logistic regression yielded consistent findings across anatomical regions (Figure 3). Only significant or borderline results are presented.

**FIGURE 3:**
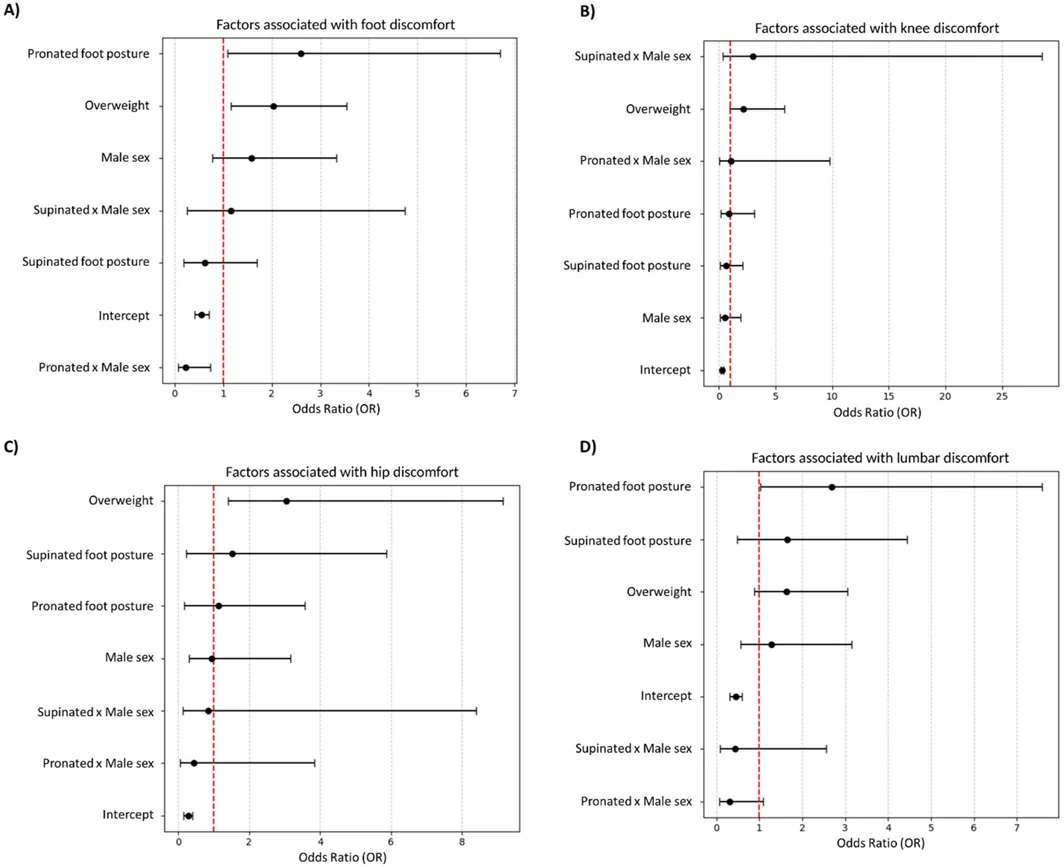
Adjusted odds ratios (95% CI) for factors associated with musculoskeletal discomfort in the foot, knee, hip, and lumbar regions. Models were fitted using Firth‐penalized logistic regression and adjusted for sex, BMI category, and Foot Posture Index (FPI). The vertical dashed line represents the null value (OR = 1). Panels (a–d) correspond to the foot, knee, hip/gluteal, and lumbar regions, respectively.

For foot discomfort, both pronated foot posture among women (OR = 2.60, 95% CI 1.09–6.71) and overweight (OR = 2.03, 95% CI 1.16–3.54) were significantly associated with higher odds of discomfort, while the pronated × male interaction indicated an attenuated association between pronation and foot discomfort in men. In the knee region, overweight displayed a borderline association with discomfort (OR = 2.18, 95% CI 0.98–5.81), and for the hip/gluteal area, overweight remained significant (OR = 3.05, 95% CI 1.41–9.16). Regarding the lumbar region, pronated foot posture was again associated with greater odds of discomfort among women (OR = 2.68, 95% CI 1.02–7.59).

Overall, the models showed region‐specific associations of pronated foot posture and overweight/obesity with discomfort, together with sex‐specific modification of the association between pronation and overall discomfort.

Results were robust to (i) the exclusion of predefined outliers and (ii) alternative grouping using “extreme” CMDQ categories. While several bivariate differences in the extreme contrast were small/borderline, the multivariable signal for pronated posture and the sex‐specific modification remained the most consistent findings across specifications.

## 4 Discussion

The present research explored the relationship between foot biomechanics and musculoskeletal discomfort in standing assembly‐line workers. All participants reported at least one musculoskeletal discomfort response in the CMDQ, which is consistent with the transient or cumulative strain induced by prolonged standing even in healthy individuals [4, 6].

Despite generally low CMDQ scores (7.5 ± 11.6 [median 2.8, IQR 0–14.2]), variability in symptom intensity allowed clear differentiation between the study‐specific analytical Low (≤ 10) and Moderate–High (≥ 11) discomfort groups. The overall distribution was markedly right‐skewed, with about one quarter of workers meeting the moderate–high criterion and only ∼6% reaching the High threshold (≥ 31), consistent with previous reports in industrial cohorts [4, 5, 6, 7]. In the anatomical distribution analysis, both groups showed similar patterns, with the feet, neck, and lumbar region as the most affected regions, aligning with evidence that prolonged standing increases lumbar load, paraspinal activation, and plantar pressures, contributing to distal and spinal discomfort [4, 6].

In the multivariable models, pronated foot posture was associated with higher odds of overall discomfort among women, whereas overweight was associated with higher odds of discomfort in the foot and hip/gluteal regions. A sex‐specific interaction indicated that this association was attenuated in men, suggesting that the association between pronated foot posture and standing‐related musculoskeletal symptoms may be more pronounced in women.

At the bivariate level, in the exploratory contrast between extremes (Low ≤ 10 vs. High ≥ 31), workers in the High discomfort group showed a greater podalic angle and slightly larger normalized heel width, whereas arch‐area differences were borderline and hallux load as well as rocker timing (ICP) did not reach significance. Moderate effect sizes suggest biomechanically meaningful differences, consistent with adaptive postural strategies under prolonged standing.

The increased podalic angle may reflect either a more pronated foot posture or an excessive external rotation of the lower limb, both of which can modify the alignment of the kinetic chain and transmit torsional stresses to the pelvis and lumbar spine. Similar associations between distal alignment and proximal loading have been reported by Ramos‐Frutos et al. [25], who observed that altered foot type changes plantar pressure distribution and proximal joint loading patterns. Structural factors such as femoral retroversion, tibial external torsion, or shortening of the hip external rotators may also contribute to an increased podalic angle and should be considered potential confounders [28, 29]. Overall, this pattern agrees with findings from Menz et al. [17] and Abbasi et al. [30], who reported an association between pronated foot function, lower‐limb malalignment and lumbar pain, particularly among women.

Although pronation is typically associated with greater medial and hallux loading during gait [9, 10, 31], the lower hallux pressure observed in symptomatic workers may reflect a compensatory unloading strategy; however, this interpretation remains hypothetical. This possible adaptation would be coherent with the greater podalic angle and heel width found, which could indicate a wider support base that may enhance stability under prolonged load [5, 8].

Although ICP was shorter in the High discomfort group, the difference did not reach statistical significance and muscle activity was not measured. One possible hypothesis is that differences in dorsiflexor control could contribute to altered rocker timing [9, 10, 32]; however, this interpretation remains speculative and should be examined in future studies using electromyographic and kinematic measures. Diminished eccentric control could accelerate the center‐of‐pressure progression, effectively shortening the heel rocker and reducing shock‐absorption efficiency [32]. These potential neuromechanical alterations would be consistent with the hypothesis that the biomechanics of prolonged standing differ from those of walking, involving compensatory postural adjustments and altered muscle activation patterns to maintain balance and mitigate discomfort [4, 5, 32].

Our findings are consistent with previous epidemiological evidence showing that prolonged occupational standing increases the risk of musculoskeletal pain. Coenen et al. [4] demonstrated that extended standing is associated with higher odds of discomfort, particularly lumbar pain, while Waters & Dick [6] and Jo et al. [33] confirmed that long periods of standing contribute to fatigue, sore feet, and lumbar symptoms. Within this context, the observed association between foot posture and discomfort aligns with the Framingham Foot Study [17] and the meta‐analysis by Abbasi et al. [30], which both indicate that pronated foot function and abnormal lower‐limb alignment predispose to lumbar discomfort, especially in women.

The sex‐specific interaction observed in our regression models indicated that the association between pronated foot posture and discomfort was weaker among men. This finding may reflect sex‐related differences in lower‐limb mechanical properties and postural control. Kubo et al. [34] reported lower stiffness and greater strain of the medial gastrocnemius tendon structures in women compared with men, while Zhang et al. [35] observed lower Achilles tendon stiffness in women. In addition, Ericksen and Gribble [36] reported greater ankle inversion–eversion laxity and lower dynamic postural control in women, and sex‐related differences in strength and lean mass have also been described [37]. These characteristics could influence how a pronated foot posture is accommodated during prolonged weight‐bearing and may contribute to differences in mechanical loading between sexes.

However, tendon properties, ligamentous laxity, muscle strength and postural control were not measured in the present study; therefore, these mechanisms should be considered hypotheses rather than direct explanations for the observed interaction. Nevertheless, the sex‐specific pattern is consistent with population‐based evidence from Menz et al. [17], who reported an association between pronated foot function and lumbar pain in women.

Taken together, these findings indicate that foot biomechanics, BMI and sex‐related factors are associated with musculoskeletal discomfort during prolonged standing. The observed FPI × sex interaction suggests that the association between pronated foot posture and discomfort differs by sex, whereas BMI showed region‐specific independent associations.

### 4.1 Strengths and Limitations

This study has several strengths. It included a relatively large and ecologically valid sample of industrial workers exposed to prolonged standing. A multimodal assessment combining the Foot Posture Index [11], baropodometry [12], and 3D foot scanning [15] provided a comprehensive evaluation of foot biomechanics. The use of Firth‐penalized logistic regression minimized small‐sample bias and improved the interpretability of associations.

Nonetheless, certain limitations should be acknowledged. The cross‐sectional design prevents causal inference, and the CMDQ, although validated in Spanish‐speaking industrial populations [19], relies on self‐reported symptoms that may involve perception bias. The moderate–high subgroup (*n* = 56) and the High subgroup (*n* = 14) were relatively small, reducing the statistical power for extreme contrasts. Only the right foot was analyzed, assuming bilateral symmetry, which may overlook subtle asymmetries. Additionally, occupational and environmental variables such as footwear, flooring type, and psychosocial factors were not recorded, despite evidence of their contribution to discomfort during standing [6, 33].

Finally, anatomical variables not included in this study, such as femoral retroversion, external tibial torsion, or hip rotator shortening, may influence the podalic angle and confound the observed associations [28, 29].

### 4.2 Implications for Occupational Practice

The identification of pronated foot posture and overweight as correlates of discomfort supports the consideration of biomechanical and anthropometric assessment within occupational health evaluations. Workers with pronated feet may benefit from supportive footwear or custom orthoses, which help redistribute plantar loads and reduce heel and lumbar pain [38]. Concurrent weight‐management strategies could further lower plantar pressures and musculoskeletal strain [39, 40].

At the organizational level, task rotation and short micro‐breaks should be promoted to reduce cumulative static load. While footrests have limited effectiveness [13], anti‐fatigue mats can decrease lumbar discomfort by enabling subtle postural adjustments and enhancing balance [5]. Preventive measures should be individualized and integrated into a broader ergonomic framework that combines personal interventions (orthoses, footwear, weight control) with environmental and scheduling adaptations [6]. Recent evidence also highlights the benefits of combining corrective and active strategies, such as custom‐made insoles and targeted exercise programs, which have been shown to reduce lower limb and back discomfort in assembly‐line workers [41]. Considering the sex‐specific differences observed, these findings suggest that sex may be relevant when considering workers with pronated foot posture in occupational‐health assessments [34, 35].

### 4.3 Future Research

Future longitudinal and interventional studies should test whether correcting pronation through orthoses or supportive footwear reduces discomfort, particularly among women in whom the association between pronation and discomfort appeared stronger [17]. Given recent evidence supporting multifactorial ergonomic programs, future trials should also examine combined interventions, such as orthoses, targeted exercise, anti‐fatigue mats, and task rotation, to assess their additive or synergistic effects [5, 6, 41].

Advanced biomechanical analyses (center‐of‐pressure trajectories, rocker timing, muscle activation) and the inclusion of contextual factors such as footwear, flooring, and psychosocial load [33] would improve our understanding of the mechanisms involved. Multicenter studies and long‐term follow‐up are warranted to enhance external validity across industrial environments.

## 5 Conclusions

In standing assembly‐line workers, pronated foot posture and overweight were associated with musculoskeletal discomfort in specific models and anatomical regions. A significant FPI × sex interaction indicated that the association between pronation and overall discomfort was attenuated in men relative to women. Exploratory differences in hallux loading and rocker timing did not reach statistical significance and should be interpreted cautiously.

These findings support further investigation of foot posture and BMI as potentially relevant factors in occupational‐health assessment and preventive strategies for workers exposed to prolonged standing.

## Author Contributions

**Alejandro‐Jesús Almenar‐Arasanz:** conceptualization, methodology, investigation, data curation, formal analysis, writing – original draft, writing – review and editing. **José‐Antonio Villalba‐Ruete:** resources, investigation, project administration, writing – review and editing. **Elisa Bravo‐Viñuales:** investigation, data curation, writing – review and editing. **Ana Vanessa Bataller‐Cervero:** conceptualization, methodology, supervision, writing – review and editing. **Juan Rabal‐Pelay:** conceptualization, methodology, project administration, supervision, writing – review and editing. **Jacobo de‐Rentería‐de‐la‐Peña:** methodology, investigation, writing – review and editing. **Luis‐Enrique Roche‐Seruendo:** methodology, supervision, writing – review and editing. **Cristina Cimarras‐Otal:** conceptualization, methodology, supervision, writing – review and editing.

## Funding

This research received no specific funding. The article processing charges (APCs) will be covered by Universidad San Jorge, Spain, through its 2026–2027 Open Access Publication Support Programme.

## Conflicts of Interest

José‐Antonio Villalba‐Ruete is employed by BSH Electrodomésticos España, the company at whose Montañana plant the study was conducted. BSH Electrodomésticos España played no role in the study design, data analysis, interpretation of results, or decision to submit the manuscript for publication. The remaining authors declare no competing interests.

## Supporting information


**Table S1:** Complete comparison of anthropometric, foot morphological and plantar pressure variables between workers with High (CMDQ ≥ 31) and Low (CMDQ ≤ 10) discomfort (extreme‐groups contrast; *n* = 198).

## Data Availability

The data that support the findings of this study are available from the corresponding author upon reasonable request.

## References

[1] S. Y. Guan , J. X. Zheng , N. B. Sam , S. Xu , Z. Shuai , and F. Pan , “Global Burden and Risk Factors of Musculoskeletal Disorders Among Adolescents and Young Adults in 204 Countries and Territories, 1990–2019,” Autoimmunity Reviews, [Internet] 22, no. 8 (August 2023): 103361: [cited 2025 April 20], https://pubmed.ncbi.nlm.nih.gov/37230312/. 10.1016/j.autrev.2023.103361 37230312

[2] V. C. W. Hoe , D. M. Urquhart , H. L. Kelsall , E. N. Zamri , and M. R. Sim , “Ergonomic Interventions for Preventing Work‐Related Musculoskeletal Disorders of the Upper Limb and Neck Among Office Workers,” Cochrane Database of Systematic Reviews, [Internet] 10, no. 10 (October 2018): [cited 2025 Apr 20], https://pubmed.ncbi.nlm.nih.gov/30350850/. 10.1002/14651858.CD008570.pub3 PMC6517177 30350850

[3] Encuesta Europea de Condiciones de Trabajo , “Datos España | INSST,” [Internet] (2021): [cited 2025 Apr 20], https://www.insst.es/documentacion/material‐tecnico/documentos‐tecnicos/encuesta‐europea‐de‐condiciones‐de‐trabajo‐2021‐datos‐espana‐2023.

[4] P. Coenen , L. Willenberg , S. Parry , et al., “Associations of Occupational Standing With Musculoskeletal Symptoms: A Systematic Review With Meta‐Analysis,” British Journal of Sports Medicine, [Internet] 52, no. 3 (February 2018): 174–181: [cited 2025 Apr 11], https://pubmed.ncbi.nlm.nih.gov/27884862/. 10.1136/bjsports-2016-096795 27884862

[5] T. B. Winberg , M. N. Glinka , K. M. Gallagher , T. B. Weaver , A. C. Laing , and J. P. Callaghan , “Anti‐Fatigue Mats Can Reduce Low Back Discomfort in Transient Pain Developers,” Applied Ergonomics, [Internet] (April 2022): [cited 2025 Apr 20];100., https://pubmed.ncbi.nlm.nih.gov/34837750/. 10.1016/j.apergo.2021.103661 34837750

[6] T. R. Waters and R. B. Dick , “Evidence of Health Risks Associated With Prolonged Standing at Work and Intervention Effectiveness,” Rehabilitation Nursing, [Internet] 40, no. 3 (May 2014): 148–165: [cited 2025 Apr 20], https://pmc.ncbi.nlm.nih.gov/articles/PMC4591921/. 10.1002/rnj.166 PMC4591921 25041875

[7] J. Rabal‐Pelay , C. Cimarras‐Otal , B. Lacárcel‐Tejero , et al., “Changes in Baropodometric Evaluation and Discomfort During the Workday in Assembly‐Line Workers,” Healthcare, [Internet] 12, no. 7 (April 2024): 761: [cited 2025 Apr 20], https://pubmed.ncbi.nlm.nih.gov/38610183/. 10.3390/healthcare12070761 PMC11011590 38610183

[8] CCOHS , “Working in a Standing Position–Basic Information,” [Internet]. [cited 2025 Apr 20], https://www.ccohs.ca/oshanswers/ergonomics/standing/standing_basic.html.

[9] A. K. Buldt , S. Forghany , K. B. Landorf , P. Levinger , G. S. Murley , and H. B. Menz , “Foot Posture Is Associated With Plantar Pressure During Gait: A Comparison of Normal, Planus and Cavus Feet,” Gait & Posture, [Internet] 62 (May 2018): 235–240: [cited 2025 Apr 20], https://pubmed.ncbi.nlm.nih.gov/29573666/. 10.1016/j.gaitpost.2018.03.005 29573666

[10] A. K. Buldt , J. J. Allan , K. B. Landorf , and H. B. Menz , “The Relationship Between Foot Posture and Plantar Pressure During Walking in Adults: A Systematic Review,” Gait & Posture, [Internet] 62 (May 2018): 56–67: [cited 2025 Apr 20], https://pubmed.ncbi.nlm.nih.gov/29524798/. 10.1016/j.gaitpost.2018.02.026 29524798

[11] A. C. Redmond , Y. Z. Crane , and H. B. Menz , “Normative Values for the Foot Posture Index,” Journal of Foot and Ankle Research, [Internet] 1, no. 1 (July 2008): 6: [cited 2025 Apr 20], https://pubmed.ncbi.nlm.nih.gov/18822155/. 10.1186/1757-1146-1-6 PMC2553778 18822155

[12] A. B. Putti , G. P. Arnold , L. A. Cochrane , and R. J. Abboud , “Normal Pressure Values and Repeatability of the Emed ST4 System,” Gait & Posture, [Internet] 27, no. 3 (April 2008): 501–505: [cited 2025 Apr 20], https://pubmed.ncbi.nlm.nih.gov/17702582/. 10.1016/j.gaitpost.2007.06.009 17702582

[13] J. H. Lee , J. Hwang , H. Park , et al., “Muscle Strength and Foot Pressure Vary Depending on the Type of Foot Pain,” Scientific Reports, [Internet] 14, no. 1 (2024): 1–8: 2024 Mar 11 [cited 2025 Apr 20], https://www.nature.com/articles/s41598‐024‐56490‐8. 10.1038/s41598-024-56490-8 PMC10928145 38467691

[14] Z. Qian , Z. Jiang , J. Wu , et al., “Morphology and Mechanical Properties of Plantar Fascia in Flexible Flatfoot: A Noninvasive in Vivo Study,” Frontiers in Bioengineering and Biotechnology, [Internet] 9 (September 2021): 727940: [cited 2025 Apr 20], www.frontiersin.org. 10.3389/fbioe.2021.727940 PMC8479101 34604187

[15] A. Ballester , A. Pierola , E. Parrilla , et al., “Fast, Portable and Low‐Cost 3D Foot Digitizers: Validity and Reliability of Measurements,” In Proceedings of 3DBODY.TECH 2017 – 8th International Conference and Exhibition on 3D Body Scanning and Processing Technologies (2017), 218–225, 10.15221/17.218.

[16] J. H. Andersen , J. P. Haahr , and P. Frost , “Risk Factors for More Severe Regional Musculoskeletal Symptoms: A Two‐Year Prospective Study of a General Working Population,” Arthritis & Rheumatism, [Internet] 56, no. 4 (April 2007): 1355–1364: [cited 2025 Apr 11], https://pubmed.ncbi.nlm.nih.gov/17393441/. 10.1002/art.22513 17393441

[17] H. B. Menz , A. B. Dufour , J. L. Riskowski , H. J. Hillstrom , and M. T. Hannan , “Foot Posture, Foot Function and Low Back Pain: The Framingham Foot Study,” Journal of Foot and Ankle Research, [Internet] 6, no. 1 (May 2013): 1: [cited 2025 Sep 23], https://jfootankleres.biomedcentral.com/articles/10.1186/1757‐1146‐6‐S1‐O27.

[18] A. Hedge , S. Morimoto , and D. Mccrobie , “Effects of Keyboard Tray Geometry on Upper Body Posture and Comfort,” Ergonomics, [Internet] 42, no. 10 (October 1999): 1333–1349: [cited 2025 Apr 20], https://pubmed.ncbi.nlm.nih.gov/10582503/. 10.1080/001401399184983 10582503

[19] J. M. Astete‐Cornejo and J. R. Asencios‐Hidalgo , “Validation of the Cornell Musculoskeletal Discomfort Questionnaires in Textile Workers in Peru,” Revista Brasileira de Medicina do Trabalho, [Internet] 21, no. 4 (2023): 01–07: [cited 2025 Apr 20], https://pubmed.ncbi.nlm.nih.gov/39132260/. 10.47626/1679-4435-2023-1029 PMC11316540 39132260

[20] R. Regal‐Ramos , “Epidemiología de la incapacidad laboral por patología osteomuscular en España: 60.000 trabajadores valorados por el Instituto Nacional de la Seguridad Social en 2019,” Medicina y Seguridad del Trabajo, [Internet] 68, no. 269 (December 2022): 209–220: [cited 2025 Sep 23], http://scielo.isciii.es/scielo.php?script=sci_arttext&pid=S0465‐546X2022000400003&lng=es&nrm=iso&tlng=es.

[21] X. Ding , Z. Guan , N. Liu , et al., “Prevalence and Risk Factors of Work‐Related Musculoskeletal Disorders Among Emerging Manufacturing Workers in Beijing, China,” Frontiers of Medicine, [Internet] 10 (2023): 1289046: [cited 2025 Sep 23], https://pmc.ncbi.nlm.nih.gov/articles/PMC10602678/. 10.3389/fmed.2023.1289046 PMC10602678 37901405

[22] A. Stewart , M. Marfell‐Jones , T. Olds , and H. de Ridder , International Standards for Anthropometric Assessment. International Society for the Advancement of Kinanthropometry (ISAK) (2011).

[23] CUergo , Musculoskeletal Discomfort Questionnaires. [Internet]: [cited 2025 Oct 29], https://ergo.human.cornell.edu/ahmsquest.html.

[24] S. Xiao , X. Zhang , L. Deng , S. Zhang , K. Cui , and W. Fu , “Relationships Between Foot Morphology and Foot Muscle Strength in Healthy Adults,” International Journal of Environmental Research and Public Health, [Internet] 17, no. 4 (February 2020): 1274: [cited 2025 Apr 22], https://www.mdpi.com/1660‐4601/17/4/1274/htm. 10.3390/ijerph17041274 PMC7068515 32079188

[25] J. A. Ramos‐Frutos , D. Oliva , I. Miguel‐Andres , et al., “Effect of Foot Type on Plantar Pressure Distribution in Healthy Mexicans: Static and Dynamic Pressure Analysis,” Physiologia, [Internet] 5, no. 3 (September 2025): 29: [cited 2025 Sep 24], https://www.mdpi.com/2673‐9488/5/3/29/htm.

[26] World Health Organization , Obesity: Preventing and Managing the Global Epidemic. Report of a WHO Consultation. WHO Technical Report Series 894 (World Health Organization, 2000). 11234459

[27] J. Cohen , Statistical Power Analysis for the Behavioral Sciences, 2nd ed. (Lawrence Erlbaum Associates, 1988).

[28] D. Hudson , “The Rotational Profile: A Study of Lower Limb Axial Torsion, Hip Rotation, and the Foot Progression Angle in Healthy Adults,” Gait & Posture, [Internet] 49 (September 2016): 426–430: [cited 2025 Oct 24], https://pubmed.ncbi.nlm.nih.gov/27513740/. 10.1016/j.gaitpost.2016.08.004 27513740

[29] D. Donati , F. Giorgi , G. Farì , L. Tarallo , F. Catani , and R. Tedeschi , “The Influence of Pelvic Tilt and Femoral Torsion on Hip Biomechanics: Implications for Clinical Assessment and Treatment,” Applied Sciences, [Internet] 14, no. 20 (October 2024): 9564: [cited 2025 Oct 24], https://www.mdpi.com/2076‐3417/14/20/9564/htm.

[30] S. Abbasi , S. H. Mousavi , and F. Khorramroo , “Association Between Lower Limb Alignment and Low Back Pain: A Systematic Review With Meta‐Analysis,” PLoS One, [Internet] 19, no. 10 (October 2024): e0311480: [cited 2025 Sep 24], https://journals.plos.org/plosone/article?id=10.1371/journal.pone.0311480. 10.1371/journal.pone.0311480 PMC11466400 39388409

[31] B. Chuckpaiwong , J. A. Nunley , N. A. Mall , and R. M. Queen , “The Effect of Foot Type on In‐Shoe Plantar Pressure During Walking and Running,” Gait & Posture, [Internet] 28, no. 3 (2008): 405–411: [cited 2025 Sep 24], https://pubmed.ncbi.nlm.nih.gov/18337103/. 10.1016/j.gaitpost.2008.01.012 18337103

[32] H. Hanawa , T. Miyazawa , K. Hirata , K. Kubota , and T. Fujino , “Contribution of Tibialis Anterior in Sit‐to‐Stand Motion: Implications for Its Role in Shifting the Center of Pressure Backward,” Journal of Functional Morphology and Kinesiology, [Internet] 10, no. 2 (May 2025): 156: [cited 2025 Oct 24], https://www.mdpi.com/2411‐5142/10/2/156/htm. 10.3390/jfmk10020156 PMC12101179 40407440

[33] H. Jo , O. B. Lim , Y. S. Ahn , S. J. Chang , and S. B. Koh , “Negative Impacts of Prolonged Standing at Work on Musculoskeletal Symptoms and Physical Fatigue: The Fifth Korean Working Conditions Survey,” Yonsei Medical Journal, [Internet] 62, no. 6 (June 2021): 510–519: [cited 2025 Sep 23], 10.3349/ymj.2021.62.6.510. PMC8149936 34027638

[34] K. Kubo , H. Kanehisa , and T. Fukunaga , “Gender Differences in the Viscoelastic Properties of Tendon Structures,” European Journal of Applied Physiology, [Internet] 88, no. 6 (2003): 520–526: [cited 2025 Oct 24], https://pubmed.ncbi.nlm.nih.gov/12560950/. 10.1007/s00421-002-0744-8 12560950

[35] X. Zhang , L. Deng , S. Xiao , L. Li , and W. Fu , “Sex Differences in the Morphological and Mechanical Properties of the Achilles Tendon,” International Journal of Environmental Research and Public Health, [Internet] 18, no. 17 (August 2021): 8974: [cited 2025 Oct 24], https://www.mdpi.com/1660‐4601/18/17/8974/htm. 10.3390/ijerph18178974 PMC8430544 34501564

[36] H. Ericksen and P. A. Gribble , “Sex Differences, Hormone Fluctuations, Ankle Stability, and Dynamic Postural Control,” Journal of Athletic Training, [Internet] 47, no. 2 (2012): 143–148: [cited 2025 Oct 24], https://pubmed.ncbi.nlm.nih.gov/22488279/. 10.4085/1062-6050-47.2.143 PMC3418125 22488279

[37] M. A. Pérez , G. P. Urrejola‐Contreras , J. Hernández , P. Silva , and M. Torres‐Banduc , “Sex Differences in Upper and Lower Strength and Their Association With Body Composition Among University Students,” Physical Activity and Nutrition, [Internet] 28, no. 3 (September 2024): 64–071: [cited 2025 Oct 24], https://pmc.ncbi.nlm.nih.gov/articles/PMC11540993/. 10.20463/pan.2024.0025 PMC11540993 39501696

[38] D. B. Irving , J. L. Cook , M. A. Young , and H. B. Menz , “Obesity and Pronated Foot Type May Increase the Risk of Chronic Plantar Heel Pain: A Matched Case‐Control Study,” BMC Musculoskeletal Disorders, [Internet] (2007): [cited 2025 Sep 24];8. , https://pubmed.ncbi.nlm.nih.gov/17506905/. 10.1186/1471-2474-8-41 PMC1884155 17506905

[39] I. Casado‐Hernández , R. Becerro‐de‐Bengoa‐Vallejo , M. Losa‐Iglesias , J. Gómez‐Salgado , D. López‐López , and J. Bayod , “Variability of the Lower Limb Symmetry Index Associated With the Gait Parameters in the Overweight Adult Population With Flatfoot: A Case‐Control Study,” Frontiers in Bioengineering and Biotechnology, [Internet] 11 (2023): 1189309: [cited 2025 Sep 24], https://pubmed.ncbi.nlm.nih.gov/37388764/. 10.3389/fbioe.2023.1189309 PMC10303102 37388764

[40] A. Jankowicz‐Szymańska , K. Wódka , M. Kołpa , and E. Mikołajczyk , “Foot Longitudinal Arches in Obese, Overweight and Normal Weight Females Who Differ in Age,” Homo, [Internet] 69, no. 1–2 (March 2018): 37–42: [cited 2025 Sep 24], https://pubmed.ncbi.nlm.nih.gov/29709300/. 10.1016/j.jchb.2018.03.001 29709300

[41] E. Mousavi , Z. Zamanian , M. Hadadi , and S. Sobhani , “Investigating the Effect of Custom‐Made Insoles and Exercises on Lower Limb and Back Discomfort in Assembly‐Line Workers in a Rubber Tire Factory: A Randomized Controlled Trial,” Human Factors and Ergonomics in Manufacturing, [Internet] 29, no. 6 (November 2019): 478–484: [cited 2025 Nov 7], 10.1002/hfm.20810.

